# FINANCIAL PREPARATION FOR RETIREMENT OF WORKING-AGE ADULTS IN HONG KONG: A PERSONALIZED PENSION PROJECTION

**DOI:** 10.1093/geroni/igad104.3039

**Published:** 2023-12-21

**Authors:** Alex Yue Feng Zhu, Kee Lee Chou

**Affiliations:** The Education University of Hong Kong, Hong Kong, Hong Kong; The Education University of Hong Kong, Hong Kong, Hong Kong

## Abstract

This study replicated and upgraded the personal pension projections among a sample of middle-aged working adults in Hong Kong (N = 400). We conducted a three-arm randomized controlled trial in which the participants were randomly assigned to either experimental condition A (a personalized pension projection), experimental condition B (a personalized pension projection plus new “identifying feasible plans to achieve the ideal retirement life” component), or the control condition. Analyzing a baseline and two follow-up tests, we found that a personalized pension projection had immediate and short-term positive effects on the participants’ financial preparation for retirement. The newly added component strengthened both effects. More specifically, the original personalized pension projection improved the participants’ financial preparation for retirement by reducing their temporal discounting. Our findings enhance understanding of the mechanism of a personalized pension projection, clarify its location within the intervention framework of retirement financial planning, and recommend multiple channels to policymakers and social workers to further upgrade and optimize a personalized pension projection and improve its effectiveness.

